# Short and long term outcome of neonatal hyperglycemia in very preterm infants: a retrospective follow-up study

**DOI:** 10.1186/1471-2431-10-52

**Published:** 2010-07-20

**Authors:** N Margreth van der Lugt, Vivianne EHJ Smits-Wintjens, Paul HT van Zwieten, Frans J Walther

**Affiliations:** 1 Division of Neonatology, Department of Pediatrics, Leiden University Medical Center, Albinusdreef 2, 2333 ZA Leiden, The Netherlands; 2 Juliana Children's Hospital, The Hague, The Netherlands

## Abstract

**Background:**

Hyperglycemia in premature infants is associated with increased morbidity and mortality, but data on long-term outcome are limited. We investigated the effects of neonatal hyperglycemia (blood glucose ≥ 10 mmol/l, treated with insulin for ≥ 12 hours) on growth and neurobehavioral outcome at 2 years of age.

**Methods:**

Retrospective follow-up study at 2 years of age among 859 infants ≤32 weeks of gestation admitted to a tertiary neonatal center between January 2002 and December 2006. Thirty-three survivors treated with insulin for hyperglycemia and 63 matched controls without hyperglycemia were evaluated at a corrected age of 2 years. Outcome measures consisted of growth (weight, length, and head circumference) and neurological and behavioural development.

**Results:**

66/859 (8%) infants ≤ 32 weeks of gestation developed hyperglycemia. Mortality during admission was 27/66 (41%) in the hyperglycemia group versus 62/793 (8%) in those without hyperglycemia (p < 0.001). Mortality was higher in infants with hyperglycemia with a birth weight ≤1,000 gram (p = 0.005) and/or gestational age of 24-28 weeks (p = 0.009) than in control infants without hyperglycemia. Sepsis was more prominent in infants with hyperglycemia and a birth weight of >1,000 gram (p = 0.002) and/or gestational age of 29-32 weeks (p = 0.009) than in control infants without hyperglycemia. Growth at 2 years of age was similar, but neurological and behavioural development was more frequently abnormal among those with neonatal hyperglycemia (p = 0.036 and 0.021 respectively).

**Conclusions:**

Mortality was higher in very preterm infants with hyperglycemia treated with insulin during the neonatal period. At 2 years of age survivors showed normal growth, but a higher incidence of neurological and behavioural problems. Better strategies to manage hyperglycemia may improve outcome of very preterm infants.

## Background

Hyperglycemia is a common problem in very preterm infants. Among extremely low birth weight infants the incidence of neonatal hyperglycemia is estimated to be between 45% and 80%[1-3]. This is possibly an underestimation as 50% of the abnormalities in glucose levels are not detected by standard intermittent sampling and the incidence is also influenced by the use of different definitions[4,5]. Accurate monitoring of blood glucose levels is clinically important, because abnormalities in glucose homeostasis can have serious short term consequences. Hyperglycemia is associated with increased mortality, which is significantly related to the duration of the hyperglycemia[6,7]. A higher incidence of retinopathy of prematurity and intraventricular hemorrhage (IVH) grade 3 and 4 has also been reported[7,8].

Prematurity is an important risk factor in the complex pathogenesis of hyperglycemia. Insulin resistance develops due to a high circulating level of inflammatory markers, cytokines and catecholamines. Consequently, glucose production in the liver is not inhibited. The pancreas needs to produce insulin to compensate, but is probably unable to do so due to immature beta-cells, leading to relative insulin deficiency[7,9,10].

Since twenty-five years continuous insulin infusion is used, nevertheless in many centers restriction of glucose intake is still the first step in treatment of hyperglycemia[5].

Comparison of these interventions has demonstrated the efficacy of continuous insulin infusion in glucose control. Infants treated with insulin had a higher glucose intake, a higher weight gain, less sepsis and an increased endogenous insulin production[11,12]. The benefit of early elective insulin therapy in the prevention and treatment of hyperglycemia has been demonstrated in critically ill adults, but is questionable in premature infants. Although early elective insulin therapy decreases hyperglycemia, it significantly increases the incidence of hypoglycemia and mortality by 28 days[13-15].

Whereas the short term effects of neonatal hyperglycemia in very preterm infants have been reported, less is known about the effects of hyperglycemia on growth and neurobehavioural outcome. The aim of this retrospective follow-up study was to determine the possible effects of hyperglycemia on growth and neurobehavioral development by comparing very preterm infants with and without a history of neonatal hyperglycemia at the corrected age of two years.

## Methods

### Study population

The study population consisted of 859 very preterm infants (≤ 32 weeks) admitted to the Neonatal Intensive Care Unit (NICU) of the Leiden University Medical Center between January 2002 and December 2006. Neonatal data and data on growth and neurobehavioural outcome at the corrected age of 2 years were collected from the charts in the tertiary neonatal center and the regional hospitals where these infants were regularly seen for follow-up. The Medical Ethics Committee of the Leiden University Medical Center did not require approval of this study because it consisted of retrospective chart review, nor did the medical ethics committee require written consent by the parents for their infant's information to be stored in the hospital database and used for research.

Hyperglycemia was defined as at least 2 blood glucose levels of ≥10.0 mmol/L (180 mg/dL) during a 12-hour period. Insulin treatment was started at 0.05 U/kg/h [14] when hyperglycemia persisted after these 12 hours, despite reduction of glucose intake to 5-6 mg/kg/min. The insulin dosage was tapered after blood glucose levels dropped to <10 mmol/L. The cohort of exposed infants consisted of all preterm infants with neonatal hyperglycemia and the unexposed cohort was a matched selection of preterm infants admitted during the same timeframe, but without hyperglycemia (and without short-lived hyperglycemia <12 hours) and insulin therapy. The infants were matched by birth weight (± 0.1 kg), gestational age (± 1 week), gender, and date of admission (a period of ± 1 year was preferred). Each exposed infant was matched to 2 unexposed controls. The exclusion criteria for both exposed and unexposed infants were major congenital anomalies and chromosomal abnormalities. The study size was determined by the number of exposed infants during the study period and the availability of matches.

### Data collection

Blood glucose levels were measured in whole blood using the glucose oxidase method. (Siemens RapidPoint 400/405 system, Siemens Healthcare Diagnostics B.V., Breda, The Netherlands). Frequency of glucose level measurements was based on clinical and laboratory findings. During the first week after birth blood glucose levels were measured at least 6 times a day, thereafter glucose levels were measured at least 3 times a day. In the acute phase of hyperglycemia, glucose levels were measured regularly with intervals of approximately 1-2 hours.

Data for demographic and perinatal characteristics as well as postnatal clinical conditions of all infants were collected from the charts and included birth weight, gestational age, length of stay (as an indicator of severity of illness), gender, exposure to prenatal and postnatal steroids, presence of chorioamnionitis, sepsis, prolonged rupture of membranes (PROM), respiratory distress syndrome (RDS)[16], bronchopulmonary dysplasia (BPD)[17], necrotizing enterocolitis (NEC)[18], cystic periventricular leukomalacia (PVL)[19] and intraventricular hemorrhage (IVH)[20,21].

For the exposed infants the mean, minimum and maximum glucose levels, glucose intake and insulin infusion rate were calculated for the first 5 days after the onset of the hyperglycemia episode or until the end of the hyperglycemia episode if the episode lasted longer than 5 days. Additionally, duration of hyperglycemia, frequency of hypoglycemic periods, duration of insulin infusion, mean serum sodium concentration, maximum daily percentage of inhaled oxygen and maximum infusion rate of inotropic drugs (dobutamine and dopamine), were recorded.

Outcome data collected at the corrected age of 2 years (± 3 months) included weight (kg), length (cm), head circumference (cm), and neurobehavioral development. Neurological outcome was classified into 3 groups: normal, mildly abnormal (detectable but not disabling abnormalities of tone and reflexes, presence of abnormal movements or asymmetry) and severely abnormal (disabling abnormalities) according to the neurological examination by Hempel[22]. Behavioural outcome was scored normal or inadequate based on the Child Behavior Checklist/2-3 (CBCL/2-3) [23] completed by the parents or orally by the clinic pediatrician.

### Statistical analysis

Data are reported as mean values ± standard deviation, numerical values or categories. Statistical analyses were performed with SPSS Version 16.0 (SPSS Inc., Chicago, IL). Numerical growth parameters were analyzed with an unpaired T-test. The categorical data for behavioural and neurological outcome were analyzed using a chi-squared test.

Subgroup analyses were performed for subgroups in gestational age (24-28 and 29-32 weeks) and birth weight (≤1,000 gram and >1,000 gram).

Potential confounders in this study were gestational age, gender, birth weight, length of stay, exposure to prenatal and postnatal steroids, presence of severe RDS, sepsis, PROM, chorioamnionitis, NEC, BPD, PVL and IVH. Confounding was prevented by matching and using multivariable regression analysis for analyzing mortality and morbidity, as well as growth, neurological and behavioural development. Infants with missing baseline characteristics were excluded from regression analyses.

## Results

**Figure 1:**
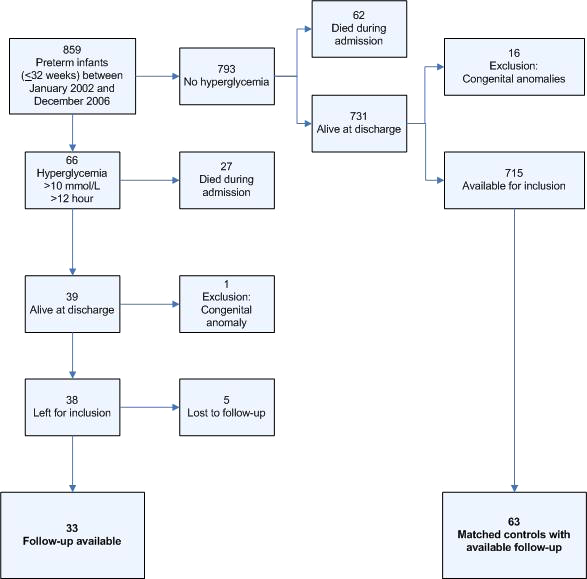
**Flow chart of included patients**.

Between January 2002 and December 2006 a total of 859 very preterm infants with a gestational age less than 33 weeks were admitted for neonatal intensive care. Mean gestational age was 29.4 ± 2.0 weeks and birth weight 1,323 ± 410 gram. Fifty-six percent was male. Prenatal steroids were administered to 63% of the mothers. Chorioamnionitis and PROM were present in 13 and 27% respectively. Twenty-nine percent of the infants developed sepsis, 6% IVH grade 3 or 4, 3% cystic PVL, 2% NEC grade 2 or 3, 19% RDS grade 3 or 4 and 14% BPD at 36 weeks postmenstrual age. Postnatal steroids were administered to 10% of the infants. Hyperglycemia occurred in 66 (8%) infants of whom 53 had a birth weight of ≤1,000 gram, the incidence in this subgroup was 25%. The mean age at onset of the hyperglycemia episode was 3.2 ± 3.7 days. Twenty-seven out of 66 infants with hyperglycemia (41%) died during admission versus 62/793 (8%) infants without hyperglycemia. Six of the remaining 39 infants with hyperglycemia were excluded from this follow-up study because of major congenital anomalies (1) and loss to follow-up (5). Emigration (1), placement in an unknown foster home (1) and no appearance at follow-up appointments (3) were the reasons for the loss to follow-up. These infants were excluded from the analysis together with their unexposed matches. For 3 exposed infants only 1 unexposed match was available. The complete follow-up population consisted of 96 infants; a flowchart can be seen in figure 1.

An overview of the baseline characteristics of included patients is given in table 1. The exposed group had a significantly longer length of stay than the unexposed group, reason why statistical correction for confounders by regression analysis was done.

**Table 1:** Baseline characteristics of very preterm infants included in the follow-up study.

Demographic and perinatal data	Exposed (n = 33)	Unexposed (n = 63)	p-value
Gestational age (weeks)	28.2 ± 2.2	28.2 ± 2.2	1.000

Birth weight (grams)	962 ± 316	1008 ± 292	0.478

Length of stay (days)	47.2 ± 32.5	25.7 ± 45.1	*0.017*

Gender (male)	21 (64%)	39 (62%)	1.000

Prenatal steroids			0.721
0 doses	12 (36%)	22 (35%)	
1 dose	13 (39%)	21 (33%)	
>1 dose	8 (24%)	20 (32%)	

Chorioamnionitis*	2 (6%)	6 (10%)	0.711

PROM**	9 (27%)	14 (22%)	0.620

**Postnatal clinical conditions**:			

Sepsis***	21 (64%)	37 (59%)	0.667

IVH grade 3/4	3 (9%)	3 (5%)	0.411

RDS grade 3/4	11 (33%)	19 (30%)	0.818

Cystic PVL	0 (0%)	2 (3%)	0.544

NEC grade 2/3	1 (3%)	0 (0%)	0.334

BPD ****	20 (61%)	25 (40%)	0.056

Postnatal steroid exposure	10 (30%)	13 (21%)	0.321

* Chorioamnionitis was defined as smelly amniotic fluid, maternal fever or signs of infection at birth.

** PROM was defined as rupture of membranes >24 hours.

*** Sepsis was defined as a positive blood culture.

**** Need for oxygen-therapy at a gestational age of 36 weeks or at discharge

Table 2 shows growth and development at 2 years of corrected age. Weight, length, head circumference were not different in the exposed cohort compared to the unexposed cohort. The incidence of an abnormal neurological and behavioural outcome was higher in the exposed group (p = 0.036 and 0.021 respectively).

**Table 2:** Growth and development at 2 years of corrected age.

Growth and outcome measures	Exposed (n = 33)	Unexposed (n = 63)	p-value
**Head circumference (cm)**	47.8 ± 1.9	48.0 ± 1.8	0.613

**Length (cm)**	85.7 ± 4.0	85.0 ± 3.9	0.410

**Weight (kg)**	11.0 ± 1.5	11.0 ± 1.3	1.000

**Neurological outcome**			*0.036*
Normal	16 (48%)	47 (75%)	
Mildly abnormal	12 (36%)	12 (19%)	
Severely abnormal	5 (15%)	4 (6%)	

**Behavioural outcome**			*0.021*

Normal	17 (52%)	48 (76%)	

Inadequate	16 (48%)	15 (24%)	

Analyses of the data on the hyperglycemia episodes (table 3) showed some significant relations. Mean and maximum glucose levels on the third and fourth day of the hyperglycemia episode correlated with mortality (mean: p = 0.046, maximum: p = 0.041). Infants with a mean glucose level > 8.0 mmol/L (144 mg/L) or a maximum glucose level > 9.5 mmol/L (171 mg/L) on the third and fourth day had a higher mortality rate (p = 0.042 and p = 0.030, respectively). All hypoglycemia episodes were brief events which were corrected quickly. Neurobehavioural outcome, growth or mortality were not influenced by the frequency of hypoglycemia periods and the duration of the hyperglycemia episode and correction for these potential confounders was therefore not necessary.

**Table 3:** Characteristics of the hyperglycemia episode in all 66 exposed infants*.

N = 66	Mean	Minimum	Maximum	SD
Age at onset of hyperglycemia (d)	3.2	1	19	3.7

Number of hypoglycemic episodes	0.3	0	6	0.9

Mean glucose level (mmol/L)	12.9	10.1	21.9	2.3

Mean glucose intake (mg/kg/min)	6.3	3.4	11.0	1.3

Mean insulin infusion rate (U/kg/h)	0.06	0.01	0.34	0.06

Duration of hyperglycemia (h)	34	12	169	25

Duration of insulin infusion (h)	129	3	754	132

* Including deaths and infants lost to follow-up.

A multivariable regression analysis on all very preterm infants admitted between January 2002 and December 2006 (n = 798 infants with complete baseline characteristics), confirmed a significant increase in mortality in the exposed cohort (p = 0.001) (Table 4). Subgroup analysis demonstrated that infants with a birth weight ≤ 1,000 gram (mean gestational age 27.3 ± 1.8 weeks) and/or a gestational age of 24-28 weeks (mean birth weight 972 ± 229 gram) also had a significant relation between hyperglycemia and mortality.

**Table 4:** Regression analysis for mortality in very preterm infants.

	Multivariate p for hyperglycemia
Total population (n = 798*)	*0.000*

Gestational age subgroups:	
24-28 weeks (n = 265)	* 0.009*
29-32 weeks (n = 533)	0.899

Birth weight subgroups	
≤1,000 gram (n = 188)	*0.005*
>1,000 gram (n = 610)	0.402

*: 61 of the 859 very preterm infants had missing baseline characteristics and were excluded from the model

In the subgroup of infants with a birth weight >1,000 gram (mean gestational age 30.1 ± 1.6 weeks) hyperglycemia was associated with an increased incidence of IVH (p = 0.025), severe RDS (p = 0.012) and sepsis (p = 0.002). Sepsis was also more prominent in a subgroup of infants with a gestational age of 29-32 weeks (p = 0.009, mean birth weight 1,503 ± 362 gram). In the regression analyses for morbidity and mortality statistical correction was done for gestational age, gender, birth weight, exposure to prenatal and postnatal steroids, presence of severe RDS, sepsis, PROM, chorioamnionitis, NEC, BPD, PVL and IVH.

## Discussion

The findings in this study indicate that hyperglycemia has no effect on long term growth. However, in addition to the already known short term effects, hyperglycemia has a distinct negative influence on neurological and behavioural outcome at 2 years of age. The observation by other investigators that hyperglycemia is a risk factor for early mortality in very preterm infants was confirmed. We found a firm association between neonatal mortality and the mean and maximum glucose levels on days 3 and 4 of the hyperglycemia episode. This association suggests that increased mortality is probably due to poorly controlled hyperglycemia. No association between mortality and duration of hyperglycemia was found. In subgroup-analyses we found relations between hyperglycemia and morbidity. Sepsis, severe RDS and IVH were more common in infants with hyperglycemia and a birth weight of >1,000 gram (mean gestational age 30.1 ± 1.6 weeks).

Hays et al. demonstrated that blood glucose concentration had significant effects on both early death and the occurrence of severe IVH[7]. To our knowledge, adverse neurological and behavioural outcome secondary to neonatal hyperglycemia has not been reported yet.

This retrospective study has several limitations. An observer-bias cannot be excluded because several physicians did the follow-up consults at the age of 2 year. To reduce the observer-bias as much as possible strict cut off points were made and the follow-up information was interpreted by only one person. In the future it would be interesting to do a prospective study, in which Bayley scores will be collected from all participants. Furthermore, it was quite difficult to match unexposed controls for date of admission within 1 year to reduce the influence of changing policies. The composition of the unexposed control group was randomly chosen and purely based on the in advance defined matching criteria. Nonetheless, unintentional selection cannot be excluded and the matched controls may not 100% represent the complete population of very preterm infants admitted to the nursery during the study period. An inevitable complicating factor in this study was the concomitant occurrence of hyperglycemia and insulin therapy, through which the individual influence of hyperglycemia or insulin therapy cannot be demonstrated. Although this study does not have the strength of a prospective follow-up study, the results will give some support and direction for the prognosis of the development of very preterm infants with hyperglycemia.

Treatment with insulin is not unequivocally associated with better outcome. The first study in adults by van den Berghe showed very promising results[15]. However, it proved to be difficult to repeat the positive results in later studies in adults, and some studies even showed negative effects. Early treatment with insulin in preterm infants (without hyperglycemia) even showed higher mortality at 28 days in the early-insulin group than in the control group[14]. The less favourable outcome in the insulin-treated group in this study could therefore also be due to the insulin therapy.

Several follow-up studies in infants with diabetes mellitus type 1 may explain our findings concerning neurobehavioural outcome. Infants with longer episodes of hyperglycemia seem to have an impaired cognitive development, though the specific impaired elements of cognitive development vary between the studies. Compared with healthy siblings, infants with hyperglycemia had a lower verbal intelligence[24]. A follow-up study conducted 2 years after the onset of type 1 diabetes suggests that hyperglycemia is associated with compromised learning- and consolidation capacities and organizational strategies. However, this follow-up study was repeated after another 4 years, at which time this relationship could not be confirmed[25,26].

## Conclusions

The results of this retrospective follow-up study suggest that hyperglycemia and insulin therapy are not only associated with increased mortality and short term morbidity, but also exert long term effects on development. Given the definition of hyperglycaemia, including the use of insulin for >12 hours, one can not assume that the association is due to poorly controlled hyperglycemia in this design, but may be the effect of insulin.

## Abbreviations

BPD: Bronchopulmonary dysplasia; IVH: Intraventricular hemorrhage; NEC: Necrotizing enterocolitis; NICU: Neonatal intensive care unit; PVL: Periventricular leukomalacia; PROM: Prolonged rupture of membranes; RDS: Respiratory distress syndrome

## Competing interests

The authors declare that they have no competing interests.

## Authors' contributions

NMvdL participated in the design of the study, collected the data, performed the statistical analysis and drafted the manuscript. VEHJS-W collected the data and helped to correct the manuscript. PHTvZ collected the data and drafted the manuscript. FJW conceived of the study, participated in its design and coordination and helped to draft and correct the manuscript. All authors read and approved the final manuscript.

## Pre-publication history

The pre-publication history for this paper can be accessed here:

http://www.biomedcentral.com/1471-2431/10/52/prepub
